# Book Reviews

**Published:** 1820-11

**Authors:** 


### CRITICAL ANALYSIS

### OF

### RECENT PUBLICATIONS, IN THE DIFFERENT BRANCHES OF MEDICINE AND SURGERY.

---

"I would have men know, that though I reprehend the easie passing over of the causes of things "by ascribing them to secret and hidden vertues and properties; (for this hath arrested and laid "asleepe all true enquiry and indications;) yet I doe not understand but that, in the practical "part of knowledge, much will be left to experience and probation, whereunto indication cannot "so fully reach: and this not only in *specie,* but in *individuo.* Yet it was well said, *Vere scire* "*esse per causas scire.*"—BACON.

---

*An Historic Sketch of the Causes, Progress, Extent, and Mortality, of the Contagious Fever epidemic in Ireland during the years* 1817, 1818, *and* 1819: *with numerous Tables, Official Documents, and Private Communications, illustrative of its general History, and of the System of Management adopted for its Suppression.* By William Harty, M.B. Physician to the King's Hospital and to the Prisons of Dublin. 8vo. pp. 512. Hodges and M'Arthur, Dublin. 1820.

An abstract analysis cannot be given of this work: the characteristics, as well as the utility of it, consist essentially in the details which it comprises; and it is the production of such details that has been the principal object of the author in its construction. A slight consideration of it will be sufficient to produce an impression that it is a work of great merit, and one which will in future times, in all probability, become of eminent utility; and a more studious perusal of it will disclose evidence of talents and industry of a rare kind, and those comprehensive views of the science and art of medicine that mark the philosophical physician.

The author, in his dedicatory Letter to the Chief Secretary to the Lord Lieutenant of Ireland, shows that he has extended his views to a subject that is too much neglected by the generality of physicians,—the relations of medicine with the policy of nations; and he also proves that he well understands the subject as far as it is connected with the matters treated of in this work, if a coincidence of his views with those of the most esteemed politicians may be considered a proof of such knowledge. He opposes the notions of those (we cannot call them disciples of MALTHUS, they have so much mistaken his doctrines), who think that the art of medicine is useless on such occasions as those contemplated in this work; that is to say, when contagious fever is ravaging a country oppressed by famine and a too-abundant population; and who believe that physicians can do nothing better than look quietly on, and behold fever "doing its business:" and he, with much energy and judgment, exposes the folly of their arguments. There is one important circumstance which, however, he has not noticed, apparently because he could not enter into sufficient details on this occasion, that refutes the policy of those who have misunderstood, in the way just mentioned, the doctrine of Malthus: it is this; that, even conceding to those politicians (which we are very ready to do) the point, that the population of a country must be confined within a certain proportion to the extent of the *means of subsistence*, (food,) the art of medicine, although it cannot directly increase the number of people,* can do what is more advantageous to the welfare of nations,—it can maintain an older and a more effective population than that which would exist without the aid of the art of medicine. For, when the population of a country is thinned by an epidemic or pestilential disease, the mortality falls in at least an equal degree, and commonly in a greater ratio, on persons of the middle period of life; thus temporarily destroying, to a certain extent, a nation's chief strength, and giving origin, for several years afterwards, to an increase of the ordinary proportion of births, and thus filling the country with comparatively useless individuals. Although medicine cannot increase the population of a coun-

---

* A man on dying, in a country where the means of subsistence are limited, and not beyond the wants of the existing population, always gives place to another, who, but for this circumstance, would not have been born, or who would die of want. The number of marriages, in regard to the population of a country, always increases considerably after extraordinary mortality, and the subsequent ratio of the births to the deaths is also greatly augmented; as SHORT long since observed, though he gave an erroneous explanation of the circumstance. The pestilence which ravaged the north of Prussia at the beginning of the present century, and which, according to SUSSMILCH, (*Gottliche Ordnung*, u. w. s. p. 327,) carried off one-third of the population, was immediately followed by such an augmentation of the proportion of births, that the ratio to the deaths was as 320 to 100; whilst the ordinary ratio is only as about 120 to 100.

try,* it can preserve alive, to a certain extent, that which exists: it may, and undoubtedly does, thus prevent the births of many children.† But this is an advantage, if the principles of Malthus are true; for, as M. Say remarks,‡ "chaque homme adulte etant un capitale accumulé qui represente toutes les avances faites pour le mettre au point où il est," it must be admitted that this capital, which is composed of annual appropriations, augments or decreases in proportion as the individual lives beyond, or approaches to, the epoch of his birth. So that (excepting the value of the labour and industry which the pregnant woman would have evinced but for her pregnancy and puerperal confinement, and the expenses attendant on this condition), the loss of a new-born child is of but little importance; whilst that of an adult destroys all the capital accumulated in him, and the interest this capital would have produced.

The island Juan Fernandez, at the time when it was discovered by modern Europeans, presented a scene of the greatest solitude in regard to animal beings, although it abounded with the most agreeable and luxurious vegetation. The navigator who then first stepped on its shores left behind him a male and female deer, which gave origin to so numerous a progeny, that, in a few years, the pasturage of the most fertile of its valleys was devastated, and furnished but an insufficient supply of aliment for its inhabitants. All the horrors of want were then experienced; the weakest animals were deprived of

---

* Even *vaccination* has not been found qualified to increase, to any considerable extent, the population of a country. Watt, by an examination of the Bills of Mortality for Glasgow from 1783 to 1813, has shown that the ratio of the mortality of children below *two years* of age has been equally great before and after the use of vaccination; the greater proportion being preserved from the fatal effects of small-pox only to die of some other disease. This fact is not peculiar to Glasgow: the same has been observed in other parts; in Pavia, for instanee, where every child is vaccinated, the mortality of children seemed to be a little lessened within the city, but it was increased in the suburbs: a circumstance also remarked by Watt at Glasgow. But, admitting, as is probably the case, if the whole of any of the nations of Europe be considered, and as it seems to be proved in France by Duvillard, that the practice of vaccination does preserve a small proportion of individuals to the adult age, it is not shown that it increases the population, as will be presently shown; but then it does, what we argue the art of medicine in general does, it produces an older and more effective population. Thus Duvillard has found that, in France, the medium period of life before the use of vaccination was 28,763 years, and since the practice of vaccination 32,256 years. At Geneva, however, according to the statement of Odier, this period was 18,511 in the sixteenth century; 23,358, in the seventeenth; and 32,295, in the middle of the eighteenth; and it is not increased, he says, by vaccination, which makes it appear likely that the calculations of Watt and Rusconi are applicable to all well-regulated cities.

† In Switzerland, for example, the ratio of births to the population has gradually lessened since the year 1620, just in the proportion in which the ratio of deaths amongst the population has lessened.

‡ In his *Traité d'Economie Politique;* an excellent work, the principles advanced in which are in general agreeable with those of Malthus.

nutriment; the strongest obtained it only in small quantity; disease came to augment the ravages of famine, and a great proportion of the deer perished to re-establish the due propotion to the diminished supply of the means of subsistence. The decrease in the number of the inhabitants permitted a considerable proportion of the island again to become covered with luxurious vegetation, and the deer again experienced no other obstacles to their multiplication than the limits of their natural fecundity. At this time, a dog and a bitch were set on-shore in this island, and soon produced a sufficiently numerous posterity to wage a terrible war with the first inhabitants. The race of the latter diminished rapidly; the strongest and most active retired to concealed places, whence they descended from time to time only as famine called them forth. In these sallies, the weakest and most imprudent deer became the prey of the dogs, but still it was only the more strong and active of the dogs that could carry them off; so that a new equilibrium became established amongst the two species of inhabitants: the weakest of the deer became the prey of the dogs, whilst the strongest of the latter were alone able to obtain the means of subsistence.

This story of the deer of the island Juan Fernandez has been adduced as an exemplification or illustration of the progress of nations in regard to population, and of the effects of epidemic or pestilential diseases on the state of nations: it is really applicable in several points, but not wholly so; for here the effect of the dogs, which have been compared with some diseases, was to produce a limited, but a vigorous and active population, amongst the deer, by destroying the weaker and less prudent of them, and leaving the more vigorous and intelligent of them to enjoy life as far as the means of subsistence would permit them, instead of being carried off by famine almost in an equal ratio with the weaker and less prudent of their fellow-species. But epidemic and pestilential diseases act in a different manner: their ravages are amongst the strong as well as the weak, and, indeed, often carry off especially the flower of a nation; as was the case with the late epidemy of Ireland, the mortality of which fell chiefly on the most vigorous of those in the most vigorous period of life; and they lead also to the other evils designated in the following passage by Dr. HARTY.

"The individual who looks to fever as a safe or efficient agent for thinning a 'redundant' population, must be grossly ignorant both of its indiscriminate selection of victims, and of its pauperising effects. It does not spare the richer, more active, more humane and useful, members of society: on the contrary, their benevolence only exposes them the more to the ravages of this disease. Amongst the poor, too, it exerts a blind and fatal influence, very different from that of the exanthemata. The latter, indeed, might with some justice perhaps be viewed as judicious agents for decimation: they spare the heads of families, and relieve the community of thousands of unproductive children, 'fruges consumere nati;' whereas, fever spares the children, and cuts off the parents, leaving the wretched offspring to fill the future ranks of mendicancy, prostitution, and crime."

It may be added to what is above stated, that the destructive agency of epidemic pestilential diseases in general does not stop at the removal of the superabundant population: it commonly extends far beyond this point; for the loss in Ireland from the late epidemy is calculated by Dr. Harty at 40,000. We do not know what increase of births on the deaths had been previously made, but it is not likely to have been proportionate. The same causes carried off 66,000 persons in Saxony in 1772, although the surplus of births to the deaths, during the whole of twenty years previously, had been but 17,000. More than one-third of the population of Prussia disappeared by the same means, in the beginning of the present century. A considerable part of Italy has more recently suffered to an almost equal extent. If we turn to less civilized nations, the destruction of human life by the same means will be found to have been still greater. Thus, 300,000 persons perished in Mexico from pestilential fever, which accompanied a famine consequent on the freezing of the corn generally on the night of the 28th of August, 1784.

The history of nations proves that a destruction even to the extent above designated is soon repaired, in regard to numbers, as we have already shown; but there is, we may repeat, a difference of very great importance between the value of these individuals and those of a state not subjected to such a cause of destruction: the most considerable proportion in the one case being constituted of unproductive children, and in the other of useful individuals.

Dr. Harty has expressed a sentiment, in the passage we have transcribed from his work, that will probably surprise some of those who read it: we mean that of the exanthemata being, "with some justice, perhaps, viewed as judicious agents for decimation." This sentiment had been already promulgated, and had excited much anger against the author of it in the minds of many of us; but it should be remembered, that the population of a country cannot possibly exceed a certain proportion to the means of subsistence; that the fecundity of human beings is so great, when limited only by natural circumstances,* as

* The number of the ancient Israelites when they entered Egypt was only 70; and when they left it, four centuries afterwards, it amounted to 603,550 men above twenty years of age, and able to bear arms, without reckoning the Levites. Feijoo relates, that one man and four women, who escaped from a shipwreck to the Isle of Pines near Madagascar in 1590, had given origin to a progeny of quickly to exceed those due bounds; and that the moral and physical evils to individuals from this excess, the easy toleration of female prostitution by our laws, and the ordinary ravages of war, are not sufficient to restrain it in its necessary limits: we have then left us only the choice between the exanthemata, or occasional attacks of famine and its concomitant pestilence, in the present state of our policy, and the present ability to produce the means of subsistence; we have only the choice between these means of effecting the necessary destruction. All civilized nations have felt this. SOLON, LYCURGUS, PLATO, and ARISTOTLE, thought the best means were in the destruction of a certain proportion of new-born children. The Romans at all times possessed opportunities for sending out colonies under favourable circumstances; and this they were constantly resorting to. Even only nine years after the second Punic war, during the whole of which they had an army of upwards of 100,000 men in the field or in garrisons, and lost immense numbers of men, they were obliged to send out a colony, to relieve, as LIVY states, their too-abundant population. The Chinese, ANDERSON says, heard with horror the news of the possibility of exterminating the small-pox: they cried out, that they did not desire to be deprived of a disease which was absolutely necessary for them, in order to avoid the painful task of exposing their new-born children to be devoured by wild beasts. Savage nations have adopted other means. The Celts, as the Chinese do even now notwithstanding the ravages of small-pox, exposed them on the banks of rivers, to be carried away by the ebbing tide. In the Isle of Formosa, women are not allowed to bring forth living children before the age of thirty-five, abortion being produced by a priestess; and it is the same circumstances, very probably, that have led other nations to anthropophagism, not only of their enemies, but of their fellow-countrymen; and induced Europeans, in the middle ages, to adopt the more cruel policy of confining a considerable proportion of the women to monasteries.

Thus, in the words of the French Malthite, M. Say, "rien ne peut accroitre la population que ce qui favorise la produc-

---

12,000 persons by the time they were discovered by the Dutch. The population of North America has been recently found to become doubled every fifteen years in the interior of the continent, but only every twenty-five years on the coasts. Here, especially in the former situation, the means of subsistence had exceeded the necessary proportion to the population. A pestilential disease having carried off a considerable proportion of the inhabitants of Iceland in the year 1707, the king of Denmark thought proper to make a law allowing a woman to have bastard children to the number of six, without any detriment to her reputation. The population increased so rapidly, that it was found necessary to abrogate the law in a few years. Had this king been better acquainted with the principles of population, he would not have given the Icelandish wits such an opportunity as they here embraced for ridiculing the eager patriotism of the young women.

tion, et rien ne peut la diminuer, au moins d'une manière permanente, que ce qui attaque les sources de la production." It has too, unfortunately, been found, that a temporary increase of the means of subsistence, is itself the means of producing famine and pestilence. A very abundant production of corn and other products of agriculture causes these articles to become so cheap,* that the cultivation of them ceases to be profitable for a few ensuing years; agriculture is consequently neglected, and scarcity is soon the necessary result: and, if in this state of matters this scarcity comes to be increased by an unfavourable season, every preparation is made for the ravages of pestilential disease. Even *plenty* itself appears to be more immediately productive of *starvation*, in certain states of a nation; of which Ireland seems to have furnished an example in the year 1815, as Dr. GRATTAN has shown, in his *Medical Report*, (pages 14 and 22.) The harvests of 1814 and 1815, Dr. Grattan observes, proved more abundant than had ever been remembered; but the sudden transition from a state of long-protracted war to that of peace so deranged the pursuits, and gave such a check to the affairs, not only of the mercantile and manufacturing classes, but also of the landed proprietors, and of the agricultural interests, that the greatest distress followed. Manufactures languished, trade decayed, all enterprize had ceased, and, without employment, the great mass of the poorer portion of the population were starving in the midst of plenty. Fever then began to prevail to a great extent amongst them.

We should apologize to our readers for having given so very imperfect a sketch of the matters appertaining to this highly important subject: it was hardly right, indeed, to enter into the consideration of it at all on this occasion, as much space would be requisite for a perspicuous development of it. We return to the work of Dr. Harty; for, although we cannot produce an analysis of its contents, we may give a catalogue and a character of the principal parts of them.

The volume commences with "preliminary observations;" the objects of which are to state the nature of the contents of the book, and the author's views in their production; to explain the way in which it has been constructed, and the sources of the documents it comprises; and to express particularly the author's obligations to Drs. BARRY of Cork, CARROLL of Limerick, BRACKEN of Waterford, WILLIAMSON of Carrick-on-Suir, MAWE of Tralee, RYAN of Kilkenny, and M'DONNEL, STEPHENSON, and THOMSON, of Belfast, for their very important contributions.

* And this cheapness increases the number of marriages, (it has, at least, been clearly shown to be the case in Sweden by WARGENTIN,) and thus leads to a redundant population, which augments the evils which soon result from it, as designated in the text.

The author then gives an "historic sketch of the commencement, progress, and duration, of the epidemic in the four provinces of Ireland." He next shows the "extent and mortality of the epidemic;" and then produces some "introductory observations to the history of the epidemic in Dublin and Cork;" describes the "progress and management of the epidemic in Dublin;" enters into a "review of the epidemic in Cork, and of the legislative measures adopted for the suppression of fever;" compares the epidemics of 1741 and 1817; and concludes the first part of the work with a general "review of the causes, medical and statistical, productive of epidemic fever in Ireland." An Appendix (occupying nearly three hundred pages) to this, consists of the documents from which the observations and inferences above alluded to were derived.

In the historic sketch of the causes, &c. of the fever, the author shows, that, for a century at least, Ireland has been afflicted with this disease to such an extent as to substantiate the statement of some of our oldest medical authorities, that fever is one of the "endemical or reigning diseases" of the island. Dr. Gerard Boate, in his Natural History of Ireland, published in 1652, enumerates, amongst the diseases to which that country is "peculiarly obnoxious," "a certain sort of malignant feavers, vulgarly called Irish agues, because they are at all times so common in Ireland." But the ordinary extent of this malady has fallen far short of the epidemics of 1741, 1801, and 1817, in regard to the extent of their prevalence over the island, and the mortality which has thence resulted. This section of the work, as well as the following respecting the extent and mortality of the epidemic in 1817, are merely statistical; but the two following sections, on the progress and management of the fever in Dublin and Cork, presents the author with an opportunity for showing the great comprehensiveness of his views, and the solidity of his judgment, as a philosophical and practical physician; and it is this part of the work, and his "general review," &c. that have drawn forth the attempt we made at the commencement of this article to express the sense we have of his merits. We have been particularly pleased with the spirit with which he exposes the evils which resulted from the bad measures of medical police that were adopted in the early stages of the epidemy, in consequence of the conflicting opinions of the ministers of the government and the physicians; and we have derived no less gratification from the consideration of his own views of the proper policy on such occasions as those alluded to in this work.

The author considers the causes of the prevalence of an epidemic fever under the distinctions of *secondary* and *primary*, which, he thinks, might with propriety be designated *medical*

and *statistical.* "The secondary or medical causes," he says, "such as contagion; crowded, filthy, ill-ventilated, apartments; neglect of personal cleanliness; are those which more immediately lead to the formation of fever: the primary or statistical, are those which give origin to the causes just mentioned, by establishing the peculiar condition and habits of a people. The former are the causes which generate fever in every country; the latter are those which distinguish one country from another, and which render the disease more or less extensively prevalent therein according to their strength and activity." These matters are discussed at length by the author in a particular manner, and in reference to the opinions which have been advanced respecting them by others. His own conclusions are indicated in the passage just transcribed; but we may nevertheless state in plainer terms, that he believes that fever of *endemic* origin may become *contagious* in its progress, when the *secondary* causes enumerated in the paragraph just alluded to exist to a certain extent; and he believes that such has been the case with respect to the late epidemy of Ireland. These doctrines he establishes, we think, in a tolerably satisfactory manner; or, to speak with more strict propriety in respect to the existing evidence, he has shown that such inferences are the most probable inferences that have hitherto been made, and, consequently, are those which should direct the policy of nations on occasions similar to that of the existence of the epidemy under consideration. At present, we have more negative than positive knowledge of the nature of the contagious matter producing such a disease as that just mentioned; and it cannot therefore be said, that the opinions of those who profess the doctrine of *exclusive contagion* in regard to the origin of the malady, are positively refuted.

In order that the opinions we have expressed respecting this work may not lead some of our readers to purchase it, to whom it will be almost devoid of utility, we should state that it contains but little that relates in a direct manner to either the practice of medicine or the pathology of fever. It is as a treatise on a subject of *medical police* that it must be characterized; and to those who are disposed to the study of this subject, by duty or inclination, we think we cannot recommend it too earnestly.

*Some farther Observations on the Subject of the proper Period for Amputating in Gun-shot Wounds; accompanied by the Official Reports of the Surgeons employed in his Majesty's Ships and Vessels at the late Battle before Algiers.* By A. Copland Hutchison, late Surgeon to the Royal Naval Hospital at Deal; Surgeon-Extraordinary to his Royal Highness the Duke of Clarence; Fellow of the Royal College of Surgeons in London, and of the Medical and Chirurgical Society; Consulting Medical Officer to the General Penitentiary, Millbank, Westminster, &c. 8vo. pp. 64. Callow, London, 1817.

This work, we have just discovered, escaped the attention of our predecessor in the management of this Journal; but, though so long a period has elapsed since the time of its publication, we could not willingly suffer to pass unnoticed such a discussion on so important a point in the practice of surgery, even were we not induced, by a sense of justice towards the author, to lay before our readers the proofs he here adduces of the propriety of the opinions he had previously advanced on this subject, and which were stated in this Journal on a former occasion. (Vol. xxxviii. p. 62.)

Some late respectable writers, we are aware, have spoken of the question considered in this work, as one that has long been decided; and one of them says, "For my part, I have not known any difference of opinion on the point: in books, it is true, it has been most amply discussed before the present generation were in existence; but, in British practice, all doubts have long been at an end." That such is not the case, is shown by the documents brought forward in the tract before us; and, whilst there are men of some place and authority who write in favour of, and medical officers who pursue, the practice here reprobated, we cannot but regard with deep interest, because of the influence they must exert, the arguments adduced against it by a surgeon of such professional rank and talents as the author of this work.

On the return to England of the squadron which had been engaged in the battle of Algiers, the author says,

"On the return of the squadron into port, I was extremely anxious to learn the particular periods at which the amputations had taken place, the nature of the wounds requiring that operation, and the different results. Deeply impressed with the momentous importance of the subject in question to the army and to the naval service, as well as to humanity in general, I lost no time in addressing a letter to the Commissioners for sick and wounded Seamen, &c. The Commissioners, with the readiness and zeal which have invariably marked their conduct when the good of the service was in view, immediately facilitated my wishes, by procuring the annexed replies to the queries contained in my letter. These documents so fully illustrate and corroborate the truth of the doctrines which I previously endeavoured to inculcate, that I hasten to lay them before the profession, in the hope of settling definitively a question so long agitated, so interesting to humanity, and of such vital consequence to the national service in future wars.

"As some of these official documents," the author adds, "are very extended, and would occupy much space, I trust I shall be excused by the writers in giving such parts only as appear to bear more directly on the subject under consideration; and I shall endeavour to give such extracts with all due candour and fairness, stating the opinions of those gentlemen who oppose or confirm the point in controversy."

The abstract here alluded to is preceded by some general remarks on the evidence comprised in those official communications; and, from the concise manner in which they are stated, it becomes necessary for us to transcribe them in detail.

"Those surgeons who may appear to have been less successful in the general result of their operations than others who pursued a different plan, and were actuated by other sentiments, cannot in justice reflect on themselves, however much it may be lamented, particularly in the sea-service, as their mode of proceeding was influenced by the opinions of gentlemen of high professional talents, and of acknowledged experience in practical surgery. The principal object of this enquiry is, to point out the dangerous tendency of those opinions, which appear to be founded on plausible reasonings deduced from effects theoretically supposed to occur, rather than the result of actual experience or just observation.

"After the perusal of these documents, it will in the first place be necessary to consider those parts in which the writers make mention of shock and alarm to the constitution on being badly wounded in battle, as the reason assigned for *deferring* amputation till these dangerous symptoms had subsided, with its results; secondly, those in which the existence of such affections is positively denied, and consequently where amputation had been *immediately* performed, with the results also; and, lastly, the statement of other surgeons of that fleet, who noticed the circumstance of a pallid countenance, feeble pulse, and tendency to syncope, occurring in some few instances immediately on the sudden loss of blood from a severe wound requiring amputation. These, I think, are the leading facts, and embrace the principal contents of the different papers under review.

"First, then, as to the shock and alarm. The only medical officer who mentions this occurrence, is the surgeon of the Impregnable; but in his report he merely states, that 'he did not amputate till the battle had terminated; and that it appeared to him, the constitution of his patients had entirely recovered from the *shock* and *alarm* usually experienced on being wounded in battle.' Now, he enters into no statement respecting the symptoms and appearances generally characterizing shock and alarm of men wounded in battle; and therefore it may be fair to infer, that such constitutional derangement was purely imaginary and hypothetical: else, why not particularly describe the attending symptoms which induced him to suspend the operations till the battle had terminated? The probability is, that his judgment was influenced and controlled by the published opinions of certain celebrated writers on this interesting subject, as he adopts precisely their phraseology, and not regulated by personal experience and observation. In this ship there were eleven amputations performed, legs and arms, but no case of double amputation occurred;* and *nine* out of that number died. Of the two that recovered, one was operated upon below the knee, and the other above the elbow.

"The authors alluded to above make much mention of *shock and alarm to the constitution;* a phrase vague, ambiguous, and undefined: but great part of this affection called shock, must no doubt be understood to consist in the state of the mind. Now, in the first place, we know that, in the ardour of battle, the same excitement of mind continues for some time after the actual infliction of the injury, however severe, as is well known to those who have witnessed the patriotic exultation of seamen, who, under the knife, have joined in the shouts of victory! The depression of mind, so unfavourable to the success of an operation, does not come on till the spirits are exhausted by pain and loss of blood. Secondly, with regard to pain itself, the same rule holds; for it is well known that the most severe wound is hardly felt at first, and that the smart terminating in agony does not come on for some time; a time which may, and should be, anticipated by operation.

"The surgeons of the Leander and Severn amputated *immediately:* all the men, excepting two, were operated upon within half an hour of their being removed to the cockpit. These gentlemen, with the surgeon of the Superb, positively deny the existence of the slightest appearance of shock and alarm to the constitution; though, on a reference to their respective papers, it will be seen that the wounds requiring amputation were, in many instances, even more severe than those which occurred on-board the Impregnable.

"In the Leander, fifteen patients underwent amputation, two of whom lost *both thighs* very high up, and one was a hip-joint case, with other severe injuries; and these were the only men that died in consequence of their wounds and the subsequent operations. We must except, however, one man, who did not belong to the ship, and who had been thrust through the stern-port from a gun-boat during the engagement; in consequence of which it was impossible to ascertain how long he had been wounded before the operation was performed. Thus eleven cases recovered after having undergone amputation. It is proper to mention, however, that two of this number died after the cicatrization of their stumps, one of phthisis pulmonalis, and the other of bilious remittent fever; but these deaths cannot be considered as militating against the principle I am so desirous to establish.

"On board the Severn, four amputations occurred, namely, one

* Some days after the battle, a second amputation occurred in one case, and the patient died.

arm and three thigh cases, all of which recovered. It will be seen, and is truly worthy of remarking, that one of these patients, far from suffering any shock or alarm, on having his leg completely carried off by a cannon-shot, very coolly and deliberately removed the handkerchief from off his neck, and wrapped it round the mangled stump, to stop the effusion of blood till he could reach the surgeon.

"The surgeons of two or three of the ships speak of the danger that sometimes occurs from the sudden loss of a quantity of blood, on being severely wounded in battle, particularly the surgeon of the Superb; but, in these cases even, the patients are stated to have been perfectly collected, and free from every thing like sensorial affection. It is here also proper to remark, that the above circumstance is observed upon in my publication already alluded to, (page 9.) The general effect of a sudden abstraction of blood from the system, is feebleness of pulse, pallid countenance, faintness, and even syncope; but all these apparently alarming symptoms are speedily removed by administering some generous cordial, as wine, diluted brandy, &c. To these occurrences we cannot justly apply the doctrine of shock and alarm to the constitution; and, in fact, need not retard the operation beyond the period necessary to rouse the dormant powers of the system by the means already pointed out, and which, at most, require not many minutes to effect.

"The greater number of wounds inflicted by contending armies, are those arising from grape-shot and musket-balls; whereas, in naval engagements, the very reverse of this is the fact in an increased ratio; for the wounds received in ships-of-war are generally produced either by the direct stroke of a cannon-shot of large dimensions, or, what is still more lamentable, by ragged fragments of timber violently rent from the planks or beams of the ship, impelled by balls infinitely larger than any ever employed in fields of battle. Wounds inflicted by splinters of wood are always more extensive, accompanied with frightful contusions and lacerations of the soft parts.

"If, then, there be any shock or alarm to the constitution, occasioned by gun-shot wounds, which should deter or interdict the army-surgeon from performing immediate amputation when such an operation is deemed indispensably necessary, how much more alarming must we expect to find this mental shock among the wounded men in naval battles, whose injuries are found much more serious and formidable, as we have fully shown in the preceding paragraph. But the truth is, that, until a late writer talked of shock and alarm, unfortunately, in all cases of gun-shot wounds, and stated the danger of amputating before the constitution had recovered from these gratuitous and hypothetical affections, no such idea had ever entered the minds of our most experienced naval surgeons.

"It has been very properly remarked by some of the medical officers employed in the expedition against Algiers, that instances of fatal hemorrhage had occurred, in consequence of the patients incautiously relaxing the tourniquets that had been applied for some time, to stop the effusion of blood till the surgeon could find leisure to operate in regular succession, or until he thought himself justified in operating.

But what man, let me ask, can bear the severe pressure of a tight ligature like the tourniquet on a wounded limb for four or six hours, without an effort to relieve himself from the painful stricture occasioned by the instrument, however short that period might be? The probability and danger of such an occurrence may, with propriety, be urged as a strong argument in favour of immediate amputation, which would effectually guard against such fatal accidents. It is indeed scarcely possible to suppose that a ligature could be continued for such a length of time, even on a perfectly sound limb, by which the circulation is impeded or totally obstructed, without being attended with serious consequences, and even a risk of ultimate gangrene; not to mention the extreme suffering of the patient.

"Since, in my recently published Observations in Surgery, I have entered pretty largely on the nature of the irritation consequent on gun-shot wounds, in which I endeavoured to demonstrate that it is not an immediate result, but an increasing affection in a greater or less degree, proportionably to the extent of the injury and the nature of the parts wounded, I do not feel it necessary to say more here, excepting briefly to observe on gun-shot fractures of the extremities requiring amputation. Let us suppose, in wounds attended by such circumstances, the bones much shattered, and the patient left for a few hours before the operation is had recourse to; surely we are not to conclude that the unhappy patient, during this painful suspense, can remain in a tranquil or quiescent state: certainly not: the probability is, that the muscles of the injured member will be attacked with spasms; the limb more or less thrown into involuntary action; and the nerves, heretofore untouched, lacerated or grated upon by ragged points, detached fragments, and sharp edges of the fractured bones, thereby increasing the irritation in an infinite degree.

"In addition to the documents before us, illustrative of the fatal consequences generally attending deferred amputations in battle, I am authorized to state, by ocular witnesses, that, in two of the ships where this doctrine was fully acted upon, several officers and seamen, so wounded as to require immediate amputation, died in the cockpit before the period had elapsed in which the surgeon felt himself justified or warranted to commence operating: two cases in particular, one of which was badly wounded a little above the knee, and the other at the ankle; and it is necessary to remark, that all the amputations performed in that ship were arm-cases."

We shall select from the documents above alluded to some other of the most important of the facts they designate, in addition to those above stated by the author, in order that our view may embrace some of the more particular points of the evidence; and if, as a matter of form, we are to state our opinion respecting the inferences drawn from them by the author, we must acknowledge them to be apparently correct, and characterized by the most dignified liberality.

The first report is that of the surgeon of the *Impregnable*, in which vessel amputation was performed in eleven cases. Only 3

two patients finally recovered. In three instances, however, the operation was not performed until some days after the injury, so that these are cases not expressly relative to the question in dispute. In the rest it was practised generally about four or five hours after the injury.* In two instances the patients died immediately, and in three about an hour, after the operation. The inferences from these cases against delay of the operation, are not, perhaps, of the most forcible kind; as, it is said, some of the patients had been sick just previous to the action; others were burned by explosions; and, besides these circumstances, the thermometer in the cockpit, after the explosions, and consequent presence of seventy burned men and boys, stood as high as from 136° to 140°. Willing to make every allowance tending to favour the reporter, we have supposed that the last circumstance was peculiar to this vessel; though it is highly probable that it was not.

Amputation was performed in five instances on-board the *Granicus:* four recovered. They were all arm-cases. Only one patient died, forty-two hours after amputation, and he had sustained "great loss of blood previous to his removal to the cockpit." Amputation was effected about five or six hours after the injuries, speaking generally, "immediately after the ship left off action." These cases, then, furnish no evidence *against* delay of the operation; but, when we state this, we do not mean to insinuate that they present evidence in its favour.

Three cases requiring amputation occurred in the *Glasgow.* Amputation was performed in the first immediately after the injury: it was an arm-case; the patient recovered. The second patient had his leg carried away at the knee, *while in the fore-top:*† he lost a good deal of blood; "but, upon administering some wine, he seemed to have recovered so as to admit of the operation, and it was performed perhaps an hour after the in-

* The author says, however, that the surgeon of the Impregnable "has particularly requested him, in a conversation he had with him, to state, that the delay in amputating on-board that ship was occasioned more by necessity than choice." Yet, we must remark, as it shows the propriety of the statement we made at the commencement of this article, that this surgeon says, in his official Report, that he "did not perform any amputation until the action was terminated; *and that it appeared to me that the constitution of my patients had entirely recovered from the shock and alarm usually experienced on being wounded in battle.*"

† The following anecdote is so interesting in a *moral* point of view, as well as in respect to the doctrine of *shock* and *alarm* in British sailors, that we cannot refrain from transcribing it. "The man was captain of the fore-top: on his leg being so wounded that only a small portion of integument kept it connected with the thigh, he, with the view of obtaining surgical aid as soon as possible, grasped a rope by which to lower himself down upon deck. When he had reached half-way, the mangled limb, over which he could not possibly have any control, became so entangled among other ropes, that he was under the necessity of raising himself upwards about three feet, that he might disengage the wounded limb with the assistance of the sound one, whilst he was still hanging by his arms; and, having accomplished this end, he descended quietly upon deck." (Private communication from the surgeon of the Glasgow to the author.)

fliction of the wound. The patient recovered. "The third was a negro, who had both arms carried away a little above the elbow by a round shot, and was also severely contused upon the chest with splinters. The operations were performed perhaps two hours after he was wounded. His principal complaint was oppression of breathing : he was put into a cot, and died in from half-an-hour to an hour afterwards, apparently from the injury of the chest."

Amputation was performed on one person only on-board the *Superb*; it was done above the knee. "The limb was amputated during the action, as soon as some intermission had taken place of wounded men coming down to the cockpit. It might be nearly two hours after the receipt of the wound. There was no shock or alarm of the system, to render it necessary to delay the operation. He has recovered." The surgeon of this vessel, Mr. ADAMSON, adds to his report of this case some judicious remarks, founded on his practical observations on former occasions, in relation to the question under consideration; and he says, "In a great number of violent gun-shot wounds of the extremities, I have never, except in those attended with excessive hemorrhage, observed any constitutional commotion which might have prevented any necessary operation from being immediately performed."

Amputation was performed once on-board the *Infernal*, about fourteen hours after the receipt of the injury. The patient was in a fair way of doing well twenty days after the operation, when he was put on-shore at Gibraltar.

"Seven cases occurred on-board the *Queen Charlotte* which required primary amputation, viz. one of the shoulder-joint, two of the arm, one of the fore-arm, two of the thigh, and one below the knee. The destruction of parts, in all those cases, was so great as to leave no doubt of the propriety of immediate amputation."

"In one case only," the reporter remarks, "did I witness that great constitutional commotion which has been said generally to follow severe wounds; and, so far from being deterred from undertaking the operation in this case by this state of commotion, I considered it an additional motive for proceeding to it without delay. The immediate consequences of the removal of the shattered limb in this case were highly satisfactory,—the commotion speedily diminished; and, in conversing with the patient some time afterwards on the subject of the operation, he expressed himself in very strong terms of the relief he had experienced from inexpressible suffering by the operation. This amputation was performed at the shoulder-joint a few minutes after the wound was received.

"One operation, as I have stated above, was performed a few minutes after the receipt of the wound. Four were performed from four to six hours; and two on the following day, about eighteen or twenty hours after they were wounded. The two last amputations were not performed sooner, in consequence of the patients withholding their consent to the operation.

"Of the amputations performed on-board the Queen Charlotte, one terminated fatally. One was discharged into his proper ship, the Granicus, three weeks after the operation, with the stump nearly healed; and five were landed at Portsmouth, all either perfectly or nearly recovered."

The fatal case was such in consequence of subsequent hemorrhage from the axillary artery, depending on ulceration of it produced by the presence of a spicula of bone rubbing against it. Here then the facts, as well as the opinions, of the reporter, Dr. DEWAR, are decidedly favourable to immediate amputation.

There were twenty wounds on-board the *Leander* which required amputation. "I amputated (says Dr. QUARRIER), during the action, and did not defer it until the constitution had recovered from the shock and alarm patients might have laboured under, none of them having exhibited that derangement of the sensorium so frequently described by authors on gun-shot wounds, and from its being my decided opinion that the knife immediately following the injury was the most effectual mode of securing the patient from such nervous or sensorial irritation." Four of the twenty died soon after the operation: one of these patients had both thighs torn off by a double-headed shot; another suffered the same injury from a cannon-ball; both had impaired constitutions. The third had also a wound in the breast; and the fourth was thrust through the stern-port of the vessel from a gun-boat; his left thigh and arm were fractured, and he was "much bruised about the chest." Here again there is an absolute want of any evidence at all adverse to immediate amputation.

Amputation was performed four times on-board the *Severn*. All the amputations were performed during the action. "I did not," says the surgeon of the ship, "perceive any symptoms of any particular shock or alarm, under which patients in that situation have been said to labour; all of them appearing uncommonly collected." All the patients recovered.

No case requiring amputation occurred on-board the *Albion* and *Hebrus*, the other vessels concerned in the engagement.

The work concludes with a copy of a letter from Dr. BAIRD to the author, in which Dr. B. says, the propriety of immediate amputation has been fully established by the evidence he witnessed, in his late visit to the wounded seamen and marines landed from Lord Exmouth's squadron, at Haslar and Plymouth hospitals. "Indeed," he says, "I cannot well conceive a more culpable practice than that of deferring an operation longer than the surgeon can give his time to perform it, leaving the patient suffering under all the irritation and pain of a shattered limb, independent of approaching and increasing inflammation; whilst an immediate amputation would free him of the former, and lessen the chance of the latter."

---

### CRITICAL ANALYSIS

OF

