# Errata

**Published:** 1820-07

**Authors:** 


					? 403, hue 4 ol the note, tor two read ten>

				

